# A phase III randomized three-arm trial of physical therapist delivered pain coping skills training for patients with total knee arthroplasty: the KASTPain protocol

**DOI:** 10.1186/1471-2474-13-149

**Published:** 2012-08-20

**Authors:** Daniel L Riddle, Francis J Keefe, Dennis Ang, Khaled J, Levent Dumenci, Mark P Jensen, Matthew J Bair, Shelby D Reed, Kurt Kroenke

**Affiliations:** 1 Departments of Physical Therapy and Orthopaedic Surgery, Virginia Commonwealth University, Richmond, VA, USA; 2 Department of Psychiatry and Behavioral Sciences, Duke Pain Prevention and Treatment Research Program, Duke University, Durham, NC, USA; 3 Section of Rheumatology, Wake Forest University, Winston-Salem, NC, USA; 4 Department of Orthopaedic Surgery, Southern Illinois University, Springfield, IL, USA; 5 Department of Social and Behavioral Health, Virginia Commonwealth University, Richmond, VA, USA; 6 Department of Rehabilitation Medicine, University of Washington, Harborview Medical Center, Seattle, WA, USA; 7 Roudebush VA Medical Center of Excellence on Implementing Evidence-Based Practice, Indianapolis, IN 46202, USA; 8 Duke Clinical Research Institute and Department of Medicine, Duke University School of Medicine, Durham, NC 27715, USA; 9 Department of Medicine, Indiana University, Indianapolis, IN, USA

**Keywords:** Knee, Arthroplasty, Coping skills, Pain, Catastrophizing

## Abstract

**Background:**

Approximately 20% of patients report persistent and disabling pain following total knee arthroplasty (TKA) despite an apparently normally functioning prosthesis. One potential risk factor for unexplained persistent pain is high levels of pain catastrophizing. We designed a three-arm trial to determine if a pain coping skills training program, delivered prior to TKA, effectively reduces function-limiting pain following the procedure in patients with high levels of pain catastrophizing.

**Methods/design:**

The trial will be conducted at four University-based sites in the US. A sample of 402 patients with high levels of pain catastrophizing will be randomly assigned to either a pain coping skills training arm, an arthritis education control arm or usual care. Pain coping skills will be delivered by physical therapists trained and supervised by clinical psychologist experts. Arthritis education will be delivered by nurses trained in the delivery of arthritis-related content. The primary outcome will be change in Western Ontario and McMaster Universities Osteoarthritis Index (WOMAC) Pain scale score 12 months following surgery. A variety of secondary clinical and economic outcomes also will be evaluated.

**Discussion:**

The trial will be conducted at four University-based sites in the US. A sample of 402 patients with high levels of pain catastrophizing will be randomly assigned to either a pain coping skills training arm, an arthritis education control arm or usual care. Pain coping skills will be delivered by physical therapists trained and supervised by clinical psychologist experts. Arthritis education will be delivered by nurses trained in the delivery of arthritis-related content. The primary outcome will be change in Western Ontario and McMaster Universities Osteoarthritis Index (WOMAC) Pain scale score 12 months following surgery. A variety of secondary clinical and economic outcomes also will be evaluated.

**Trial Registration:**

NCT01620983

## Background

Pain is the predominant complaint of patients seeking total knee arthroplasty (TKA) [1-3], a common and generally effective procedure for patients with advanced knee arthritis [1]. Kurtz and colleagues estimated that in 2010, surgeons would perform over 700,000 TKA procedures in the US and projections suggest 3.5 million TKAs annually by 2030 [4]. Cost data for TKAs also are impressive. Mean procedural and rehabilitation costs per patient, reported in 2006 dollars, were approximately $20,700 per primary surgery and $24,500 per revision surgery [5] plus significant patient costs incurred over 12 months following surgery [6].

Serious early surgical complications such as pulmonary embolism or joint infection lead to poor outcomes. However, the incidence of these adverse events is very low - approximately 2% of all surgeries. Failure of the prosthesis is typically a late complication occurring years following the surgery and accounts for approximately 5% of poor outcomes [7]. The large majority of “poor” outcomes following knee arthroplasty are attributed to disabling pain and impaired function that is not related to early complications or prosthetic loosening. In large patient samples, improvements in pain or function scores have consistently been on the order of 40% to 60% relative to baseline, from 6 months to 2 years postoperatively [8-16].

However, some patients respond poorly to the surgery. For example, Puolakka and colleagues found that 36% of 433 patients reported daily disturbing pain four months or more after surgery [17,18]. Hawker et al. reported similar estimates 2 to 7 years following arthroplasty [19]. Only a third of patients report no functional problems following surgery [20] and approximately 20% report dissatisfaction with their functional ability a year or more after surgery [21]. Functional deficits following surgery are observed in a wide range of activities, with up to 40% of patients still requiring the use of an assistive device to ambulate [19]. Most recently, Beswick and colleagues reported in a systematic review that 20% of patients in high quality cohort studies reported persistent function-limiting pain six months or more following TKA [22].

Disabling pain and reduced function is a large and as yet unsolved problem that has a dramatic impact on quality of life and productivity. For example, revision surgery rates are influenced by persistent pain and impaired function. Roberts and colleagues conducted a survival analysis of 4,400 patients with knee arthroplasty and found that 15 years following surgery, a total of 239 knees required revision and up to 35% were for unexplained pain. Extrapolating to current estimates, as many as 35% of 55,000 revision arthroplasty surgeries in the US in 2010 may be attributable to unexplained persistent pain and subsequent poor function [23].

A barrier to improving postoperative outcomes is that traditionally, knee arthroplasty has been presumed to be a highly effective procedure. TKA perioperative protocols have historically not incorporated routine screening for patients at-risk for post-surgery persistent pain or compromised function because this area has not been scientifically investigated. This culture has strong potential for change, however, because recent research has begun to acknowledge that unexplained poor outcomes occur [22]. Predictors of these poor outcomes following knee arthroplasty have been identified [24-27]. Among the most consistent and powerful psychological predictors of poor outcome following knee arthroplasty is pain catastrophizing [17,26,28-32]. Individuals who catastrophize tend to ruminate about pain, magnify the threat value of pain and feel helpless when dealing with pain [33,34].

Additional impetus to address the issue of persistent pain was proposed by the National Institutes of Health (NIH). An NIH consensus panel was convened to review existing evidence regarding the use of knee arthroplasty surgery and to make recommendations for future research to improve the care for these patients [1]. The panel placed high priority on research examining the impact of perioperative interventions for these patients. Our trial will specifically target this research need. If a high-quality trial demonstrates that pain coping skills training is successful at improving outcomes for at-risk patients with poor pain coping, and is cost effective*,* current clinical practice paradigms could be significantly improved.

The primary aim of our study is to assess the efficacy of a physical therapist-delivered pain coping skills training program in reducing knee pain and improving function. Our two primary hypotheses are that in patients scheduled for knee arthroplasty and with comorbid pain catastrophizing: (1) Pain coping skills training is more effective than arthritis education in decreasing knee pain during functional activities, and (2) Pain coping skills training is more effective than usual care in decreasing knee pain during functional activities. We also will examine two sets of secondary hypotheses: (1) Pain coping skills training is more effective than arthritis education or usual care in improving self-reported function, physical performance, pain intensity, pain catastrophizing, and patient global ratings of improvement and (2) Pain coping skills training will reduce direct medical costs and indirect (i.e. patient time) costs relative to arthritis education and usual care. We hypothesize that, in addition to accounting for costs associated with pain coping skills training, the intervention will be cost saving or cost-effective relative to arthritis care education and usual care as measured by the incremental cost per quality-adjusted life-year. We will also determine if treatment benefits are mediated by changes in pain catastrophizing: We hypothesize that treatment-related changes in pain catastrophizing will mediate treatment-related improvements in pain and self-reported function during recovery.

## Method/design

### Study design

**Figure 1:**
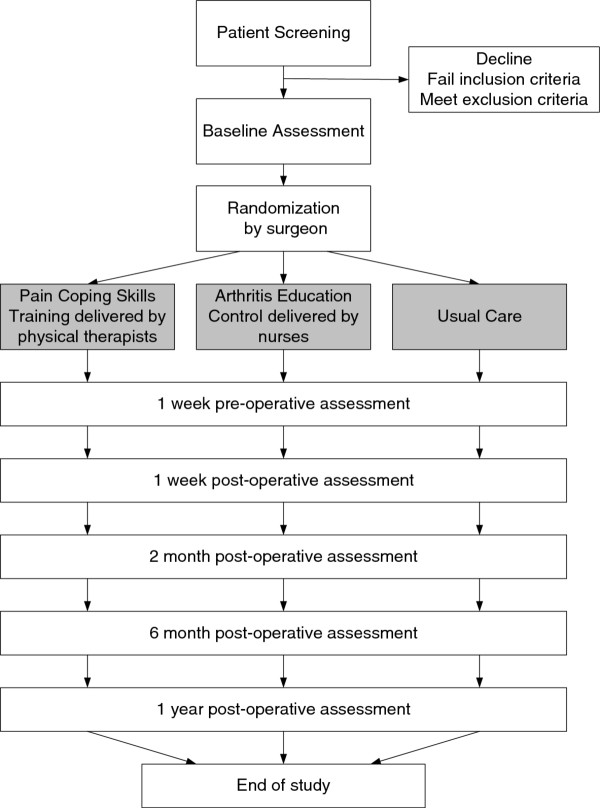
Legend: The figure illustrates the flow of subjects through the trial.

The KASTPain Trial is a three-arm randomized trial (see Figure 1) funded by the National Institute of Arthritis, Musculoskeletal and Skin Diseases/National Institutes of Health (1UM1AR062800). The investigators, research assistants assessing outcomes, and patients assigned to two of the three study arms will be blinded to group assignment. Potential subjects will be informed that they will be randomly assigned to one of two different educational treatments or usual care. They will likely not know whether they will be receiving the treatment with the hypothesized key ingredient (pain coping skills training). The protocol conforms to the CONSORT guidelines for nonpharmacologic interventions [35]. Human subjects approval for the study has been obtained from the Virginia Commonwealth University Institutional Review Board (HM14326).

### Study population

We will recruit 402 patients who consult with participating orthopaedic surgeons in one of four sites for TKA. These sites are: (1) Virginia Commonwealth University in Richmond, Virginia; (2) Southern Illinois University in Springfield, Illinois; (3) Duke University in Durham, North Carolina; and (4) Wake Forest University, Winston-Salem North Carolina. To be eligible for participation all patients must: (1) be able to read and speak English and provide informed consent; (2) be 45 years or older; (3) have a diagnosis of osteoarthritis as determined by the patients’ orthopaedic surgeons; (4) be scheduled for an elective unilateral total knee arthroplasty no sooner than 2 weeks or later than 8 weeks from the time of recruitment; and (5) score of ≥ 16 on the Pain Catastrophizing Scale and ≥ 5 on the WOMAC Pain scale. Scores of 16 or greater on the Pain Catastrophizing Scale suggest at least a moderately severe pain catastrophizing while scores of ≥ 5 on the WOMAC Pain Scale suggests greater than minor function limiting pain. Patients will be excluded if they: (1) are scheduled for revision arthroplasty surgery; (2) are unable to or decline consent for study participation; (3) have a self-reported diagnosis of inflammatory arthritis (i.e. rheumatoid arthritis, psoriatic arthritis, systemic lupus erythematosis, ankylosing spondylitis); (4) have TKA scheduled because of a fracture, malignancy or an infection; (5) are scheduled for bilateral TKA; (6) are scheduled for unicompartmental arthroplasty; (7) report plans to undergo hip or knee arthroplasty within one year after current TKA; (8) underwent contralateral knee arthroplasty surgery or hip arthroplasty surgery within 1 year of currently planned surgery; or (9) score 20 or higher on the Patient Health Questionnaire-8 (PHQ-8) depression screener which indicates likely severe depression [36,37]. Pregnancy is an “automatic” exclusion because women who are pregnant are excluded by their physicians from TKA.

### Procedures

All patients scheduled for TKA with orthopaedic surgeons who conduct at least 50 TKAs per year at one of the four university-based study sites will potentially be eligible for study. We will only recruit surgeons who conduct ≥ 50 TKAs per year because of the potential influence of TKA volume on outcome [38]. Following an opt-out period, each patient will be contacted by phone to assess for eligibility after providing verbal consent for screening. All patients enrolled in the study will attend an in-person session to read and sign the consent form and complete pre-operative data collection procedures. In addition, all patients will complete a series of performance-based measures which include a 6-minute walk test [39] and the short physical performance battery [40], which includes a series of tests of walking speed, balance and strength.

Two in-person data collection sessions will occur for each patient, one at the baseline visit and the other at 1 year following TKA. In addition, staff that are blinded to group assignment will contact patients by phone at 4 different time points; 1 week prior to and 1 week following TKA, 2 months, 6 months following TKA (see Figure 1) to collect outcome data. Key measures obtained at each time point will be the Pain Catastrophizing Scale, the WOMAC Scale, and a knee pain rating scale [42].

### Randomization and allocation concealment

All eligible subjects will be randomized following baseline data collection into the pain coping skills arm, the arthritis education control arm or the usual care arm. Patients will be blinded to study hypotheses and will be informed that the study is examining the potential benefits of two approaches to improve outcomes as compared to usual care. The study statistician will prepare a random numbers table to permit randomization in permuted block sizes of 3 and 6, stratified by surgeon. To conceal randomization, assignment will occur via the web interface only after all baseline data are collected. The physical therapists delivering the pain coping skills and the nurses delivering the arthritis education cannot be blinded.

### Interventions

Participants randomized to one of the two treatment arms will receive eight 1-hour sessions of one-on-one instruction to be delivered over a 2-month period beginning approximately 2 weeks prior to surgery and ending 6 weeks following surgery. The first session will be delivered in-person and subsequent sessions will be delivered via the telephone. Telephone-based behavioral interventions for pain management have been shown to be effective [42,43].

### Pain coping skills

The 8-session pain coping skills training (CST) will be delivered over a 2-month period by physical therapists. This number of sessions of CST has been shown to be effective in several behavioral trials [44-50]. Study physical therapists must have practiced for at least 2 years and reported experience in the treatment of patients with TKA.

The (CST) protocol will: (1) provide a rationale for the coping skills intervention; (2) train patients in cognitive restructuring as well as a variety of skills that provide patients with opportunities to observe the impact of coping skills on changes in negative pain-related cognitions typical of pain catastrophizing (i.e. thoughts related to pain rumination, pain magnification, and helplessness in the face of pain); and (3) provide training in strategies for enhancing maintenance of gain following treatment. Melzack and Wall's gate control model of pain will be used to help patients reconceptualize their pain and emphasize their own abilities to control pain. The gate control model highlights the role that thoughts, feelings, and behaviors can play in influencing the transmission of noxious signals from the periphery to the brain [51]. Training in pain coping skills will be described as a way of changing thoughts, feelings, and behaviors that contribute to pain.

Using techniques drawn from cognitive therapy, patients will be taught how to identify irrational, maladaptive, and catastrophic pain-related thoughts and to replace these with alternative, rational, reassuring and adaptive thoughts [52]. Self-instructional training will be used to teach patients how to use calming self-statements as a way of coping with pain flares [53]. Activity-rest cycling and pleasant activity scheduling [54-56] will be used to help patients increase their activity level and observe the resultant impact on their pain-related cognitions. Activity-rest cycling teaches patients to target activities they tend to overdo (e.g. prolonged standing or walking while shopping) and learn to break these activities into periods of moderate activity (e.g. 30 minutes of shopping) followed by a limited rest break (e.g. 5 minutes of rest). Over time, the goal is to help patients raise their activity level by increasing the length of their activity and decreasing the length of their rest periods. In pleasant activity scheduling, patients learn how to identify activities they enjoy doing (e.g. reading, doing hobbies, and visiting friends) or that give them a sense of mastery (learning how to do something new such as typing or a new language) and then set and record weekly activity goals.

Patients also will be trained in three attention diversion methods that can be used to alter negative pain related cognitions: relaxation, imagery, and distraction. Progressive relaxation training [57] will help patients learn to concentrate on muscle tension signals and use them as cues to relax. Patients will be taught how to use pleasant imagery as a way to alter their pain-related thought patterns and foster relaxation [58]. Distraction training will involve training in how to focus on physical stimuli (e.g. a photograph or picture of a nature scene) or auditory stimuli (e.g. listening to music) when experiencing increased pain [58]. Using relapse prevention methods, each patient will develop a written maintenance plan that includes the list of pain coping skills learned during the study, potential high risk situations, early warning signs of setbacks, and plans about how the patient might apply these skills in dealing with future setbacks and challenges. Table 1 highlights the key elements of the CST protocol.

**Table 1:** Components of the pain coping skills intervention

**Training objective**	**Coping skill training methods**
Altering Cognitions to Change Pain Catastrophizing	Cognitive Restructuring, Self-Instructional Training
Altering Activity Patterns To Change Pain Catastrophizing	Activity-Rest Cycling, Goal Setting
Using Attention Diversion to Change Pain Catastrophizing	Relaxation Training, Imagery, Distraction
Enhancing Maintenance	Relapse Prevention Training

### Arthritis education

Patients randomly assigned to the arthritis education arm will receive detailed information from a registered or licensed practical nurse educator about osteoarthritis and its treatment. The arthritis education intervention will control for participation in a trial, time and clinician attention. The arthritis education sessions will use a presentation and discussion format similar to that originally described by Lorig for arthritis education [59-62]. Figures and discussion sessions will present information on the nature of arthritis, the post-operative course of knee arthroplasty, treatment of osteoarthritis, the role of exercise, joint protection and making future treatment decisions.

This general approach to an arthritis education comparison condition has been used successfully in many behavioral studies and in several trials conducted by Keefe and Jensen [44-50,62]. An education comparison condition is a credible treatment and allows us to test whether the "pain coping" component of the experimental intervention is the specific treatment element that reduces pain and improves function over that seen in patients who receive a similar dose of time and attention from a health professional, but no training in pain coping skills.

### Usual care

We have added a “usual care” group to determine real world effects of pain coping skills. Patients in the usual care group will only receive care that they would have routinely received had they not been entered in the study. All patients in this group will undergo the same data collection procedures as patients in the other two treatment arms with the exception of the eight treatment sessions.

### Co-interventions

Patients are routinely prescribed medications for pain control and are referred for physical therapy following TKA. Data regarding medication and extent of rehabilitation therapies will be tracked throughout the study period and will be adjusted for, if necessary, in the analyses.

### Treatment adherence and fidelity

Because multiple study sites will participate and increase the risk of low fidelity due to differing approaches in applying the interventions, the research team will take a number of steps to ensure that the treatment protocols for CST as well as arthritis education are delivered uniformly by all treatment providers involved in the study. First, all pain coping skills training will be delivered by physical therapists with at least 2 years of clinical experience including the treatment of patients following TKA. Second, all physical therapists will receive coping skills training in a 2-day workshop delivered by clinical psychologist experts at Duke University. Experienced nurse educators will all be trained in 2-day workshops by DLR, a physical therapist with 30 years of experience and research in arthritis and rehabilitation, to provide the arthritis education. Third, all physical therapists and nurse educators will be provided with detailed treatment manuals and the treatment strategies will be taught through didactic instruction, illustrations of techniques from model cases, and role-play of common scenarios. Fourth, we will institute several “best practices” to enhance and monitor treatment fidelity of the pain coping skills training and arthritis education which include: (1) careful attention to the study design; (2) intensive training; (3) role playing of treatment skills enactment during training; and (4) on-line documentation of treatment delivery. All sessions for both groups will be audiotaped and supervisors or investigators will review tapes periodically for each physical therapist and nurse educator during the study. Remedial training will be provided for those clinicians who deviate from established protocol.

### Outcomes

The primary outcome measure and endpoint will be the WOMAC Pain score measured 1 year following knee arthroplasty [63-67]. The WOMAC has been studied extensively and its scales have been shown to be reliable and valid for quantifying the extent of both pain and disability in patients undergoing knee arthroplasty [16,68,69]. Secondary outcome measures will be the WOMAC Disability scale, Pain Catastrophizing Scale (PCS) [33,70,71], a verbal pain rating scale [72,73], and a patient global rating of change scale measured on a numerical rating scale from -5 (vastly worse) to +5 (completely recovered) [74-76]. All outcome measures are summarized in Table 2. Outcomes will be measured pre-operatively, 2 months, 6 months and 1 year post-surgery. To assess for potential mediating effects of pain catastrophizing on outcomes, the PCS and WOMAC scales will be administered one week prior to and 1 week following TKA. Two performance-based measures, the 6-minute walk test [39,77] and the Short Physical Performance Battery will be assessed at baseline and 1 year post-surgery [40].

**Table 2:** Summary of primary and secondary outcome and cost measure

**Measure**	**Specific instrument**
Primary outcome Measure	
Function related pain	WOMAC Pain Scale (3.1 Likert version)
Secondary Outcome Measures	
Self-reported function	WOMAC Physical Function Scale (3.1 Likert version)
Pain catastrophizing	Pain Catastrophizing Scale
Pain intensity	Numeric Pain Rating Scale
Global rating	Global rating of change scale
Walk test	6-minute walk test
Physical Performance	Short Physical Performance Battery
Cost Measures	
Employment status	Self-report
Healthcare visits	Self-report
Emergency room or urgent care visits	Self-report
Inpatient admissions	Self-report
Health Status	EQ-5D-5L

### Power considerations and data analysis

The primary endpoint will be the WOMAC Pain score at 1 year. Changes of 2 or more points in the 20-point WOMAC Pain scale indicate clinically important differences in pain-related function between individual patients [78-81]. We powered the KASTPain study to detect a difference of at least 2 points between the mean pain scores in the pain coping skills group and the arthritis education group [82].

Using a two-sided, two-group t-test of differences in means with alpha set at 0.05 and assuming the intervention difference minus the arthritis education control difference is at least 2 WOMAC Pain points, a sample size of 107 in each group will provide 91% power to detect this difference, assuming that the common standard deviation is 4.34 (based on pilot work). This corresponds to a moderate effect size of 0.46 which is consistent with the effect of other behavioral interventions for knee arthritis [83-85]. This sample size also provides 80% power to detect a 20% difference between groups in the proportion of patients with a 50% or greater improvements in WOMAC pain relative to baseline scores [9,10,12-15,86]. This effect is equivalent to an odds ratio of 2.25 and a number needed to treat (NNT) of 5 [87].

The required sample size is 321 (107 patients per arm) for the planned three-arm trial. Based on our pilot study, we expect 5% attrition due to early drop-out resulting from cancelled surgery. Patients who undergo surgery and drop out due to lack of interest, unrelated medical illness or loss of follow-up will be included in the intent-to-treat analysis. Our previous work with similar types of patients suggests that loss to follow-up will likely be approximately 20% one year following surgery [14,30]. Therefore, 402 patients (134 patients per study arm) will need to be enrolled in the trial. The accrual and retention numbers will be monitored during the study to assure that sample size estimates are reached. Stratification by surgeon is expected to reduce outcome variability and thereby increase power.

Intention to treat (ITT) will be the primary approach for all analyses. Because we expect that some patients will provide consent for our study but may opt out of surgery for a variety of reasons, we will compare the ITT analysis to the results for patients who actually undergo surgery (i.e., attrition analysis). For the primary analysis, the effect of treatment will be assessed using linear mixed models with time as a repeated factor. The model will account for correlation over time within participants, correlations within surgeons, and baseline covariates. Surgical approach, complications, medication and physical therapy use will be assessed for potential effects. Restricted maximum likelihood method (REML), which uses all available data, will be used to estimate the linear mixed model. REML is the default option in multiple software packages for mixed models including SAS and Mplus. Wothke has shown that no other missing data handling method, regardless of missing data mechanism, performs better [88].

Estimates of the effect of pain coping skills training will be obtained by constructing linear contrasts to compare the outcome at each of the key time points (baseline, 2 months, 6 months and 1 year) between the pain coping skills group and the two control groups, with adjustment for the other variables. Analyses of the secondary outcome variables will be conducted using a similar approach. For the analyses using dichotomous outcomes of ≥50% improvement at 6 months and 12 months, generalized linear models with logistic regression will be used to compare the proportion of patients with ≥50% improvement after adjustment for covariates.

As an additional secondary analysis, we also will examine the effects of potential moderators. Moderators are patient characteristics that predict treatment effects [89]. Additional psychosocial issues may influence treatment effects in knee arthroplasty. Potential moderators for patients with knee arthroplasty who may particularly benefit from pain coping skills training are the following: treatment expectations prior to surgery [90-92], self efficacy [93,94], extent of social support [9,95] and depression [28]. Because our study is not powered to test for these potential moderators, they will be assessed in the context of hypothesis generation rather than hypothesis testing and only for the primary outcome of WOMAC Pain at 1 year. This analysis will be performed by including a two-way Moderator X Treatment group interaction term in the mixed models analyses. We will assess whether these variables independently predict those patients who are more likely to respond to pain coping skills training versus arthritis education or usual care. Pain coping skills training emphasizes pain coping strategies, unlike usual care or arthritis education and because of this emphasis, we suspect that coping strategies training will be particularly effective in patients with adverse psychosocial characteristics. Knowing whether any of these potential moderators actually predict response to treatment may aid in better identifying individuals who are more likely to respond to the intervention.

### Economic evaluation

#### Medical resource use and total costs

We will also compare medical resource use and mean total costs incurred over the one-year follow-up period in the KASTPain study between patients randomized to coping skills training vs. arthritis education vs. usual care. Counts of medical resource use will include inpatient stays, outpatient visits to physicians, physical therapists and other providers, and days of pain medication. Sources for unit costs assigned to medical resource use will include average Medicare payments for inpatient care and outpatient services and average wholesale prices published in the Red Book for medications. The TEAM-HF Costing Tool will be used to estimate intervention costs by accounting for providers’ time spent delivering the study intervention [96]. Total costs will consist of direct medical costs associated with the study interventions and costs associated with TKA, rehabilitation, complications and pain management as well as patients’ time costs associated with receipt of the study interventions. Total costs, from the societal perspective, will consist of direct medical costs, intervention-related costs and indirect costs. From the health care system (or payer) perspective, indirect costs will be excluded. Sensitivity analyses will be performed to evaluate the impact of scaling up the intervention (i.e. more patients per session; fixed costs allocated over more patients) and methodological choices for cost assignment [97,98].

#### Cost-Effectiveness analysis

A cost-effectiveness analysis will also be performed. Incremental cost-effectiveness ratios (ICER) will be calculated as the difference in the mean costs per patient between study arms divided by the difference in estimated quality-adjusted life-years (QALYS). Mean costs will be estimated as described above. QALYs will be estimated using patient-level utility estimates derived from the EQ-5D, administered at baseline, 2 months, 6 months and 1 year [99]. Because the study interventions represent fixed-, one-time costs while the benefits of the interventions may last beyond the one year follow-up period in the KASTPain study, we will conduct sensitivity analyses that extrapolate differences in utilities measured at the end of follow-up over 3, 5, and 10 years, assuming that the interventions do not differentially impact survival. If we observe statistically significant differences in costs (not including costs for the intervention or initial surgery) between treatment arms at 12 months, we will extrapolate treatment-specific cost estimates over 3, 5, and 10 years, consistent with the time period for QALYs. We will evaluate uncertainty by estimating 95% CIs for estimates of costs, QALYs and measures of cost-effectiveness (i.e. ICERs or net health benefits [100]) using nonparametric bootstrapping.

## Discussion

Our study will be the first to examine the efficacy of a pain coping skills training intervention delivered by physical therapists to patients at risk for poor outcome following orthopaedic surgery. The use of physical therapists as interventionists is innovative because the intervention is traditionally delivered by clinical psychologists. However, given the limited availability of clinical psychologists and the large volume of TKA procedures, physical therapists routinely treat patients prior to and following TKA and are optimally positioned to provide coping skills training.

Previous work by our group [30] and others [28,29,32,101] has identified a TKA patient subgroup at risk for poor outcome. Patients with high levels of pain catastrophizing have been consistently shown to have a higher rate of persistent pain and compromised function compared to non-catastrophizers [28,102,103]. Current clinical paradigms do not discuss formal identification of patients at-risk for poor outcome due to ineffective pain coping [104,105] nor do these paradigms address the use of perioperative interventions to reduce poor outcome risk. Our trial will provide high-quality evidence to potentially challenge this practice paradigm. If the KASTPain intervention is effective, the research will provide strong evidence to consider augmenting this traditional approach with a scalable intervention that could improve pain and functioning for thousands of patients with TKA who are at high risk for poor outcomes.

## Abbreviations

TKA: Total knee arthroplasty; WOMAC: Western Ontario and McMaster Universities Osteoarthritis Index.; NIH: National Institutes of Health; PHQ-8: Patient Health Questionnaire-8; CST: Pain coping skills training; PCS: Pain Catastrophizing Scale; NNT: Number needed to treat; ITT: Intention to treat; REML: Restricted maximum likelihood method; ICER: Incremental cost effectiveness ratio; QALYS: Quality adjusted life years.

## Competing interests

Drs. Riddle, Keefe Ang, Saleh, Dumenci, Jensen, Bair, Reed and Kroenke have no competing interests to declare.

## Authors’ contributions

All authors participated in the conception and design of the protocol and helped to draft the manuscript. All authors read and approved the final manuscript.

## Pre-publication history

The pre-publication history for this paper can be accessed here:

http://www.biomedcentral.com/1471-2474/13/149/prepub
